# The Occlusal Side Effects of Mandibular Advancement Device Therapy in Adult Sleep Apnea Patients: A Systematic Review

**DOI:** 10.7759/cureus.48682

**Published:** 2023-11-12

**Authors:** Avni Rana, Anjana Raut, Anmol Mathur

**Affiliations:** 1 Prosthodontics, Kalinga Institute of Dental Sciences, Kalinga Institute of Industrial Technology Deemed to be University, Bhubaneswar, IND; 2 Public Health Dentistry, Dr. D.Y. Patil Vidyapeeth, Pune, IND

**Keywords:** obstructive sleep apnea (osa), side effect, mandibular advancement devices (mads), long-term therapy, dental occlusion

## Abstract

Mandibular advancement devices (MADs) remain a popular non-invasive treatment modality for the management of obstructive sleep apnea (OSA). However, the occlusal side effects from long-term therapy may result in poor patient compliance and patient drop-outs. Hence, knowledge of the possible side effects of these devices on occlusion is necessary. This article attempts to systematically review the evidence available in support of the possible long-term effects of mandibular advancement therapy on occlusion in adult sleep apnea patients.

A detailed search was conducted for unpublished and published literature and their references in various electronic databases. A grey literature search was also performed. Studies until June 30, 2022, were selected. Randomized controlled trials, non-randomized trials, and cohort studies investigating the occlusal side effects of MADs for the treatment of snoring or OSA with a follow-up of at least four years were included. Study selection, data extraction, and risk of bias assessment were performed individually and in duplicate. The risk of bias was assessed by Cochrane tools for randomized and non-randomized studies. Fourteen studies were selected for the final qualitative analysis.

The side effects reported were upper incisor retroclination, lower incisor proclination, decreased overjet and overbite, and change in the total occlusal contact area.

The review concludes that long-term MAD therapy has statistically and clinically significant effects on occlusion.

## Introduction and background

Obstructive sleep apnea (OSA), a potentially fatal disorder, is one of the most common disorders in the wide spectrum of sleep-associated disordered breathing. It has become a serious public health issue due to its enormous incidence, estimated to affect approximately a billion people globally, thereby causing severe morbidity and mortality while imposing significant economic and societal implications on healthcare systems and society at large [1]. It is characterized by repeated, episodic partial or total, upper airway obstruction in sleep, despite continued respiratory efforts [2]. Untreated sleep apnea has been reported to cause morbid effects on the affected individual’s health [3]. An increased incidence of hypertension, coronary artery disease, and stroke among other cardiovascular symptoms has been reported in OSA subjects, along with the risk of diabetes mellitus and other endocrinal and neurological disorders. Oral appliances have been widely employed for OSA management as an independent treatment option and also as an adjuvant to other modalities.

These appliances have a varying number of designs and mechanisms but the most common ones are the mandibular advancement splint (MAS) or mandibular advancement device (MAD) and the tongue retaining device (TRD) [4].

MAD advances the mandible anteriorly by creating a traction force, subsequently increasing the muscle tension in the genioglossus and supra and infra-hyoid areas, thereby increasing the pharyngeal air space [5]. Long-term MAD use has been reported to have widened the pharynx, causing thinning and shortening of the soft palate and greater head flexion [6]. The majority of the patients have reported a good benefit versus risk ratio after long-term OSA therapy [7]. Discomfort in the teeth and jaws, increased saliva production and experience of an abnormal bite are some problems that become prominent after long-term use [7,8]. The incidence of temporomandibular disorders may be associated with a longer treatment with mandibular repositioning appliance (MRA) [9]. Since these devices take anchorage from the dental components, inadvertent forces to the teeth and subsequently the patient’s occlusion is inevitable. This raises a concern to understand their effect on occlusion with long-term use. This study aims to systematically review the present scientific evidence pointing toward the possible long-term effects on occlusion of adult patients with clinically diagnosed snoring or OSA undergoing MAD therapy.

## Review

Methodology

The study followed the Preferred Reporting Items for Systematic Reviews and Meta-Analyses (PRISMA) guidelines [10] and was registered in the International Prospective Register of Systematic Reviews (PROSPERO) under the number CRD42022383646. The PICOS (Population, Intervention, Comparison/Control, Outcome, Study Design) principle was applied to formulate a focused research question, i.e., “What effect do mandibular advancement devices have on occlusion in adult sleep apnea patients undergoing a long-term appliance therapy?” and define the selection criteria for the planned systematic review. Participants were adult patients (18 years and above) diagnosed with OSA or primary snoring, the intervention was MAD therapy for four years or more, and the control intervention was other treatments, i.e., continuous positive airway pressure, placebo, uvulopalatopharyngoplasty (UPPP), or before treatment measures. The primary outcome was side effects on occlusion assessed by clinical examination, cephalometric analysis, and study model measurements. The review considered only randomized control trials (RCTs), non-randomized clinical trials, and cohort studies published in the English language. Studies on oral appliances other than MADs or combined therapy with MADs and TRDs, patient-perceived or non-specific occlusal side effects, and where the device was given after any surgical correction were excluded.

Information Sources and Search Strategy

A systematic literature review was carried out by conducting a search in the following electronic databases: PubMed, Scopus, Web of Science, ScienceDirect, ProQuest, SAGE Journals, and Cochrane Library. Searches were conducted from the inception of the database to June 2022. Related abstracts and conference proceedings were also screened. A grey literature search was also performed. Customized search strategies were constructed for each electronic database. English language restriction was applied in the search strategy. The MeSH (Medical Subject Headings) terms and related keywords like "obstructive sleep apnea," "mandibular advancement devices," occlusion," "side effects," etc. were paired together with the use of Boolean operators “AND” and “OR” to build keywords for the database search strategy. The database search results are depicted in Table 1.

**Table 1:** Database search results

Database	No. of results
PubMed	156
Scopus	11
Web of Science	890
Cochrane Database	7
ScienceDirect	19
ProQuest	20
Sage Journals	66
Grey literature	9
Total no. of articles	1178

Study Selection

**Figure 1:**
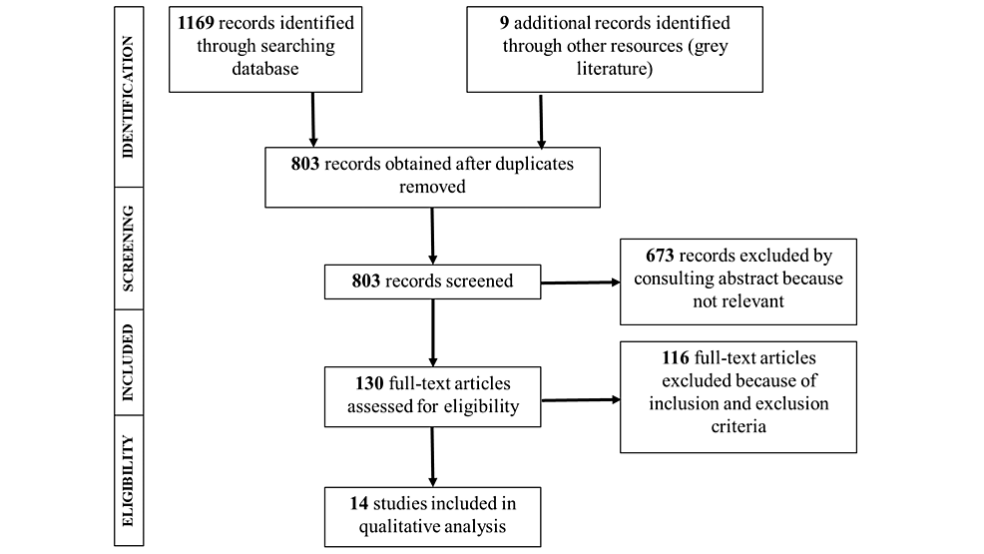
PRISMA flow diagram for study selection PRISMA: Preferred Reporting Items for Systematic Reviews and Meta-Analyses.

The collected data were manually searched for the identification and exclusion of duplicated research, and the final studies were selected adhering to the PRISMA checklist, which has been depicted in a flowchart in Figure 1. Study selection was conducted by two authors, both independently and in duplicate. Any selection process discrepancies were resolved by the third author. After identifying all potentially relevant studies from the titles, their abstracts were screened to eliminate any non-eligible articles. The references of the selected list of articles were hand-searched and backtracked to find any additional studies that could have been missed previously. Among these selected full-text articles, the duplicates were removed. Finally, the full-text articles were read independently by the same two authors and the selection was done according to the selection criteria mentioned above.

Data Items and Collection

For each study selected, first author name and publication year, study design, method of assessing the occlusal changes (clinical examination, cephalometric analysis, dental study model assessment), MAD type and protrusion amount, sample size, mean population age, follow-up period, and authors’ main conclusion were collected. The data were collected by two reviewers independently and then compared for the accuracy of data collection. Discussions were made with a third reviewer to resolve conflicts and disagreements if any. These data were converted in the form of a table to compile the individual study results.

Risk of Bias/Quality Assessment in Individual Studies

To evaluate the risk of bias in individual RCT studies, the Cochrane Collaboration’s tool for assessing the risk of bias in randomized trials (Rob 2.0) [11] was employed. Each domain judgment and thus the overall risk of bias was categorized as "low," "high," or showing "some concerns" as per the tool guidelines. For the risk of bias assessment in individual cohort studies, the Risk of Bias in Non-randomized Studies of Interventions tool (ROBINS-I) [12] was utilized. The possible results of each bias domain and their overall risk of bias were categorized as "low risk," "moderate risk," "serious risk," "critical risk," and "no information" as per the tool guidelines. Two review authors individually assessed each article and then compared the findings. Any disagreements in the assessment were discussed with the third review author and then resolved.

Results

Initially, the search resulted in 1178 articles. After reviewing their titles and abstracts, the elimination of irrelevant and duplicate articles resulted in 130 full-text articles, which were then assessed for eligibility. A total of 14 articles were finally selected for qualitative analysis based on the inclusion/exclusion criteria mentioned above. Among these, two studies were RCTs [13,14], six had a prospective cohort design [15-20], and six had a retrospective cohort design [21-26].

Risk of Bias of Individual Studies

**Figure 2:**
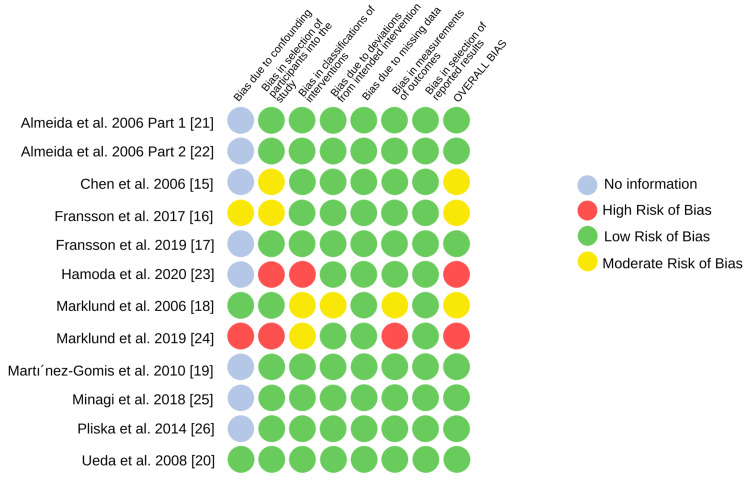
Risk of bias in individual non-randomized cohort studies (ROBINS-I)

**Figure 3:**
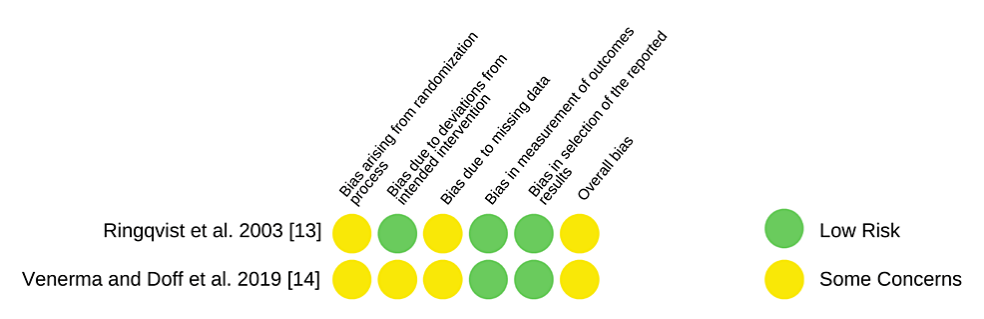
Risk of bias in randomized trials - Cochrane Collaboration’s RoB 2.0 tool

The risk of bias assessment of the selected RCTs and non-randomized cohort studies is depicted in Figures 2, 3, respectively. Both the RCTs [13,14] resulted in some concerns regarding the overall bias risk. Upon assessment, seven studies [17,19-22,24,26] had a low risk of bias, and the quality of the study could be compared to a meticulously conducted randomized trial. Two studies [23,24] reported a serious overall risk of bias and three studies [15,16,18] showed a moderate risk of bias. No bias was seen in the reported result selection domain of the non-randomized studies.

Results of Individual Studies

Table 2 represents a compilation of the individual study results. The follow-up period for the reviewed investigations ranged between four and 12 years. The study population studied in Almeida et al. part 1 [21] and part 2 [22] were the same, hence their results were considered as different reports but from the same conducted study. Five studies [13,17,21,23,25] reported the dental changes using cephalograms, six [14,16,20,22,24,26] used dental study models, one study [15] employed a three-dimensional computerized study model, researchers in one study [19] did clinical examination for dental changes assessment, and one group of researchers [18] employed both study models and clinical examination for assessment. The selected study subjects for the majority of the investigations [15-18,20-23,25,26] were patients diagnosed with snoring or OSA. The amount of mandibular protrusion also varied across different studies (50-75%).

**Table 2:** Description of the studies included in the systematic review OSA: obstructive sleep apnea; MAD: mandibular advancement device; OJ: overjet; OB: overbite; OCA: occlusal contact area; TMD: temporomandibular joint disorders; MMA: methyl methacrylate; UPPP: uvulopalatopharyngoplasty; 3D: three-dimensional.

Author	Study design	Assessment method	Sample size	Mean age of sample population (years)	Subject type	Description of type of MAD	Amount of protrusion	Follow-up period	Author's main conclusion
Almeida et al. [21]	Retrospective	Lateral cephalogram	71	49.7 ± 9.7 years	Snoring/OSA	Klearway Appliance (thermoplastic, two-piece, titratable)	66.60%	7.3 ± 2.1 years	Significant decrease in OJ, OB, and inter-incisal angle, significant maxillary incisor retroclination, significant mandibular incisor proclination, extrusion, and distal tipping of maxillary molars.
Almeida et al. [22]	Retrospective	Dental study models	70	50.0 ± 9.7	Snoring/OSA	Klearway Appliance (thermoplastic, two-piece, titratable)	66.60%	7.4 ± 2.2 years	85.7% of subjects showed favorable changes in occlusion, the Class I craniofacial subgroup was more likely to develop unfavorable occlusal changes, significant changes in inter-molar and inter-canine distances and arch length in the mandible; more stable occlusion in the maxilla.
Chen et al. [15]	Prospective	3D computerized study model	70	50.0 ± 9.6	Snoring/OSA	Titratable oral appliance	Unclear	88.4 ± 26.7 months	Long-term appliance therapy changed the posterior teeth relationship anteroposteriorly, decreased OJ, and opened the bite.
Fransson et al. [16]	Prospective	Dental study models	74	55	Snoring/OSA	Heat-cured MMA monobloc	75% of maximum protrusion	10 years	Both unfavorable and favorable occlusal changes resulted after nocturnal MAD therapy for long periods. Posterior teeth retrusion and decreased OB and OJ were seen.
Fransson et al. [17]	Prospective	Lateral cephalogram	65	-	Snoring/OSA	Monobloc of heat-cured MMA	≥75% of maximum protrusion	10 years	OB and OJ decreased as a consequence of mandibular incisor proclination and mandibular incisor retroclination.
Hamoda et al. [23]	Retrospective	Lateral cephalogram	62	49 ± 8.6	Snoring/mild-to-moderate OSA	Klearway or SomnoDent - semi-rigid thermoplastic material	66.60%	12.6 ± 3.9 years	Progressive dental changes – OB and OJ decreased, retroclined mandibular incisors, and proclined maxillary incisors.
Marklund et al. [18]	Prospective	Clinical examination and dental casts	51	51	Snoring/OSA	Soft elastomer or hard acrylic, single piece, non-titratable	4-6 mm forward	5.4 ± 0.8 years	OB changes decreased eventually but OJ continuously decreased. Large OJ reduction was prevented by soft appliance therapy in deep bite cases.
Marklund et al. [24]	Retrospective	Dental study models	38	64 years	OSA	Unclear	Unclear	9.5 years	Overjet and overbite decreased significantly, lower molars repositioned anteriorly, significantly increased lower anterior teeth irregularity, no increase in the spacing between the teeth
Martínez-Gomis et al. [19]	Prospective	Clinical examination	15 (with follow-up)	54.1 ± 8.7	OSA	2 lateral telescopic attachments connecting 2 full coverage splints made of acrylic	70% of maximum protrusion	58 months	OB and OJ decreased significantly. TMD prevalence was not significant.
Minagi et al. [25]	Retrospective	Lateral cephalogram	64	57.7 ± 14.2	Snoring/OSA	Acrylic monobloc	60-70% of maximum protrusion	4.3 ± 2.1 years	OJ, OB, and inclination of the mandibular incisors reduced significantly.
Pliska et al. [26]	Retrospective	Dental study models	77	47.5 ± 10.2	Snoring/mild-to-severe OSA	Klearway Appliance (thermoplastic, two-piece, titratable)	66.60%	11.1 ± 2.8 years	Dental occlusion changes (mandibular crowding, OB, and OJ) were progressive and significant clinically.
Ringqvist et al. [13]	Prospective, randomized controlled trial	Lateral cephalogram	30 (after 4 year follow-up in the MAD group)	-	Mild-to-moderate OSA	Heat-cured MMA single piece monobloc	50% of maximum protrusion	51 months	Clinically irrelevant skeletal and dental changes were seen, and UPPP and MAD groups had no difference in any measured variable.
Ueda et al. [20]	Prospective	Dental study models	45	49.2 ± 8.7	Snoring/OSA	Klearway Appliance (thermoplastic, two-piece, titratable)	66.60%	6.8 ± 2.4 years	Significant changes in total OCA - decreased OCA in the first molars on both the left and the right sides and increased OCA in the second molars.
Venema et al. [14]	Randomized control trial	Dental study models	14 (with MAD evaluated at 10 year follow-up)	49 ± 10	OSA	TAP appliance (Thorton Adjustable Positioner) duo block MAD	50% of maximum protrusion	10 years	OJ and OB changes were pronounced and significant.

Most of the included studies concluded that long-term therapy with mandibular advancements significantly decreased the overjet and overbite as compared to the baseline values [15-19,21,23-26]. Some studies [17,21,23,24] had a consistent conclusion of retroclination of maxillary incisors and proclination of the mandibular incisors after long-term appliance use. One study [20] derived no inferences for overjet and overbite but found a pronounced and significant change in the total occlusal contact area (OCA) post-long-term appliance therapy. Ringqvist et al. reported small and insignificant dental changes and no difference between any variables of the two controlled interventions studied [13].

Discussion

The present systematic review included 14 investigations showing dental changes after a long-term mandibular advancement appliance therapy. Among oral appliances, only the studies conducted on mandibular advancement appliances were selected so that the occlusal side effects could be correlated with the appliance design and mechanism. This review included studies with a follow-up period of more than four years to deduce the possible occlusal changes that may occur after long-term appliance therapy. A drawback of long-term studies is the high patient drop-out rates, which could be attributed to discomfort caused by the appliance. This further incorporates an added risk of bias due to a deviation from the intervention intended initially. The inconsistency in the follow-up period itself is an inherent bias while comparing individual study conclusions. However, they independently suggest a strong correlation with the amount of change in occlusal parameters.

Not many RCTs comparing the side effects of MADs have been followed up in the literature previously. Hence the chosen study designs for the review were both RCTs and cohort studies. The selection of non-randomized studies demonstrates a shortcoming of the present review since the quality of evidence is low as compared to a well-conducted RCT. In the assessed RCTs, the moderate risk of bias was mainly attributed to the weakly determined randomization procedure and high drop-out rates in both studies. The study conducted by Venema et al. [14] was reported to have added concerns of deviation from the intervention intended. The overall serious risk of bias in the investigations done by Hamoda et al. [23] and Marklund [24] is accredited to the high risk of selection bias in both studies. Since the intervention was not clearly classified, it contributed an added risk in the investigation by Hamoda et al. [23]. A serious confounding bias and a significant risk in outcome measurement contributed to the low research quality in Marklund et al.’s study [24]. Most of the selected studies have reported no information regarding the control of confounding factors in their study design [15-17,19,21-23,25,26]. This ambiguity in the reported confounders represents a vulnerability of the selected studies to an increased risk of bias. The confounding factors that could, in their presence, majorly affect the occlusion in long-term treatment outcomes of MADs, may include the periodontal status of teeth, metabolic diseases, systemic disorders, and associated drug intake among others.

The oral devices prescribed in the selected individual studies are of both titratable and non-titratable nature. Moreover, the amount of mandibular protrusion for each type of MAD varies from 50% to 75% of the maximum possible mandibular protrusion. This unpredictability of the mandibular protrusion across studies induces a heterogeneity in the true values of the treatment side effect. The material used for appliance fabrication also varies across different studies and within studies, which could impart more bias in the intervention selection and the subsequent observed dental changes. Marklund et al. [18] reported that patients using soft elastomeric MADs showed fewer changes in the overjet. The method of evaluating the dental effects is also different in all studies. The study models could be unreliable if the reproduction of the intraoral details is not done efficiently.

A significant decrease in the overjet and overbite was a consistent finding across all studies in this review, though the reported values of the change varied. Fransson et al. [16] found a decrease of 1.8 mm and 1.5 mm in overjet and overbite, respectively. A mean reduction in overjet of 1.5 mm and a mean overbite reduction of 0.7 mm in the mandibular protruding device (MPD) group was observed by Fransson et al. [17]. A 0.6 mm decrease in both the overjet and overbite of the study population was reported in Marklund’s research [18]. In another study, they also reported a median reduction of 1.6. mm in the overjet and 0.7 mm in the overbite [24]. Almeida et al. reported a significantly altered maxilla-mandibular relationship with an overjet decrease of 2.6 mm, a decrease in overbite of 2.8 mm, and a 4.1° decreased inter-incisal angle [21]. A group of researchers [18] reported that the reduction in the amount of overjet was less in infrequent users than in patients using the appliance on a continuous basis. Pliska et al. [26] also reported significant changes in the upper and lower arch relations and decreased overjet and overbite. They concluded that an increased frequency of wearing the appliance is positively related to an increased change in the occlusion. In the above-discussed literature, the decrease in overjet and overbite can be accredited to the pressure exerted by the appliances on the teeth. This device is in intimate contact with the anterior teeth and hence transmits a palatal force on the maxillary anterior in an attempt to counteract the protrusive force component. This may be responsible for the significant maxillary incisor retroclination. This change in incisor angulation can be linked to decreased overjet and overbite and the subsequent open bite in patients.

Almeida et al. [21] reported a significant incisor retroclination, distal molar tipping, and molar extrusion of 0.5 mm in maxilla. Their study showed a significant incisor proclination along with mesial tipping and 0.7 mm extrusion of the molars in the mandible. Fransson et al. [17] reported findings similar to the other studies indicating a significant retroclination of maxillary incisors and proclination of mandibular incisors, which were confluent with those of Minagi et al. [25]. Hamoda and colleagues [23] also reported statistically significant anterior tipping of mandibular incisors and posterior tipping of the maxillary incisors in their lateral cephalogram-based study. For nearly two decades of treatment, a constant progression was observed in the proclination of the upper incisors. Mandibular incisors were also observed to be progressively proclining, but their proclination rate was seen to be decreasing on continuous follow-ups and eventually ceased after treatment. The observed occlusal changes had no skeletal contribution, indicating that the malocclusion was attributed predominantly to dental movements and not the postural changes of the mandible.

Ueda et al. [20] found that 87% of their study population showed a significant change in the OCA. Martínez-Gomis et al. [19] reported that the five-year MAD treatment significantly decreased the number of contacts on the posterior occlusal table. Almeida et al. [22] found that MAD therapy commonly resulted in an open bite or edge-to-edge relationship of the premolars. MAD therapy in the long term can cause mesial tipping of the second molars (distal cusp) and cause occlusal interferences, subsequently causing an open bite in the premolar/molar region, and thus the occlusal contact changes. Since these appliances have full coverage and envelop the entire occlusal table, along with their property of mandibular protrusion, they create a posterior open bite and subsequent occlusal changes after long-term use. The change in the occlusal contacts has also been reported subjectively by another researcher [24]. This would be indicative of the fact that the patient experiences a changed pattern of chewing and may report masticatory inefficiency.

A mesial shift trend in the occlusion on subsequent follow-up appointments in long-term treatment was found to be significant in independently conducted research [16,18,22,23]. Complete occlusal coverage and mechanical loading of all teeth while wearing the device could be an explanation for maxillary molar distalization and mandibular molar movement distally. Another study also reported significant posterior open bite and anterior crossbite [26]. There was also a reported significant expansion of the mandibular arch with increased inter-canine and inter-molar distance. These findings were consistent with the reports published by other authors [18,19,22]. Small and statistically significant but clinically insignificant dental and skeletal changes were exclusive to the study conducted by another investigation [13]. They reported changes only in the vertical position of the maxillary incisors. This was attributed to the appliance being constructed with no acrylic covering the incisors but with a minor allowance for teeth movement.

Because the majority of changes reported are in the anterior teeth, the present systematic review suggests a need for studies on mandibular protrusion appliance design to evaluate if the pressure on the anterior teeth and subsequent occlusal changes could be avoided. Since the majority of studies systematically reviewed were of a non-randomized cohort design, the quality of literature evidence was not good. The included studies in this review presented an enormous amount of heterogeneity owing to the varying study designs, different devices used, the selection bias, the lack of identifying the confounders, and the high rate of patient drop-outs. Consequently, a meta-analysis was not performed hence definitive conclusions could not be drawn to quantify the results.

## Conclusions

The results of this systematic review pointed to the following conclusions: (1) long-term oral appliance therapy has a significant effect on occlusion in patients with OSA; (2) the dental changes induced by the mandibular protrusion devices include a tendency for developing a mesio-occlusion, an anterior open bite, and also hampering the occlusal contacts. Since MAD treatment is progressive in nature, a regular follow-up is necessary. Even though these appliances alter the occlusion, their role in improving the symptoms of OSA outweighs this risk.
